# Endogenous growth hormone in human retinal ganglion cells correlates with cell survival

**Published:** 2009-05-04

**Authors:** Esmond J. Sanders, Eve Parker, Steve Harvey

**Affiliations:** Department of Physiology, University of Alberta, Edmonton, Alberta, Canada

## Abstract

**Purpose:**

Locally synthesized growth hormone (GH) may act as a survival factor in several tissues. Experimental studies with chick retinal ganglion cells (RGCs) suggest that GH, synthesized within the developing retina, may have autocrine or paracrine roles in the regulation of the waves of cell death characteristic of RGC differentiation. There is also evidence that GH may have a similar neuroprotective function in the rat retina, however, there is no information concerning such a role in the human retina. In this paper we extended our earlier work by determining whether the local expression of retinal GH correlates with RGC apoptosis in human retinas.

**Methods:**

In the absence of experimental approaches to survival factor function in the human retina, we have used a correlative immunocytochemical technique to determine how the expression of GH relates to cell death in RGCs. We used sections of human retinas, taken postmortem, that we double-labeled for GH and apoptotic cell death using terminal deoxynucleotidyl transferase dUTP nick end labeling (TUNEL).

**Results:**

We found that approximately 35% of cells in the ganglion cell layer (GCL) were both GH positive and GH receptor (GHR) positive, and that GH colocalized with GHR in these cells. However, none of the apoptotic cells in the GCL were GH immunoreactive. Labeling of sections with the RGC marker, synuclein, indicated that at least 95% of the cells in the GCL were RGCs, leading us to conclude that the majority of the cells that we have examined in the GCL are RGCs.

**Conclusions:**

The results are consistent with the earlier proposal, based on in vivo and in vitro experimental chick embryo studies, that GH promotes survival in adult human RGCs.

## Introduction

We have previously shown that growth hormone (GH) is present in the retina and vitreous of the developing chick embryo [1], in perinatal and adult rats [2], and in the adult human [3]. In these tissues, GH is synthesized in the retinal ganglion cells (RGCs) from where it is secreted and stored in the vitreous [4]. During chick embryo development, this GH appears in the retina and vitreous before the functional differentiation of the pituitary gland, and it appears to act locally in the retina as an autocrine or paracrine growth and differentiation factor promoting cell survival [5,6]. Like other paracrine growth factors, which act during development [7], GH appears to activate specific intracellular antiapoptotic signaling pathways in the RGCs leading to cell survival [8,9]. It is thus thought that retinal GH is an endogenous neuroprotective agent that may regulate programmed cell death in the developing retina [10].

Since GH is present in adult human and mammalian retinas [2,3], we have sought to determine whether there is any evidence that GH might also be neuroprotective in human RGCs in which it is present. This is of potential significance since glaucoma is a disease of RGC degeneration in the elderly [11], which may be ameliorated by exogenous neurotrophic factors such as brain-derived neurotrophic factor (BDNF [12]). A positive correlation between the presence of GH and RGC survival might therefore provide a rationale for considering GH as a possible therapeutic RGC neuroprotective agent.

In the present study, we have examined sections of human retinas for the presence of GH in the cells of the RGC layer, and correlated this with the occurrence of RGC death using the method of terminal deoxynucleotidyl transferase dUTP nick end-labeling (TUNEL). We found that none of the cells in the ganglion cell layer (GCL) that expressed GH were TUNEL-positive, suggesting that the presence of GH in the RGCs is likely involved in the promotion of cell survival.

## Methods

Paraffin wax-embedded sections of human retinas, taken postmortem, were obtained from Dr. Yeni Yucel, Department of Ophthalmology and Vision Sciences, University of Toronto (Toronto, Ontario). Experiments were performed after approval by the Health Research Ethics Board of the University of Alberta. The formalin-fixed specimens used in this study were taken from three different cadavers: a 74-year-old male (cause of death: myocardial infarction; specimen fixed 11 h postmortem); an 81-year-old male (cause of death: myocardial infarction; specimen fixed 2 h postmortem); and a 69-year-old male (cause of death: amyotrophic lateral sclerosis; specimen fixed 13 h postmortem). Results were consistent between sections from each of these individuals.

For immunocytochemical double labeling, the sections were dewaxed and blocked for 1 h in 3% BSA (BSA) and then incubated overnight, at 4 °C, with a 1:200 dilution of rabbit anti-human GH (provided by Dr. A. F. Parlow, National Hormone and Peptide Program, Torrance, CA), followed by a 2 h incubation in a 1:200 dilution of goat-anti-rabbit secondary antibody that was conjugated to Alexa-Fluor 488 (green; Invitrogen, Carlsbad, CA). All washings mentioned in this paragraph were done in phosphate buffered saline (PBS). The PBS contained: NaCl, 7.2 g/l; Na_2_HPO_4_, 1.48 g/l; KH_2_PO_4_, 0.43 g/l. After washing, sections were then incubated overnight in 30 μg/ml Mab 263, a mouse anti-human GH receptor (GHR) monoclonal antibody (AbCam, Cambridge, MA). Sections were then washed and incubated for 2 h with 1:200 goat-anti-mouse Alexa Fluor 594 (red; Invitrogen). After washing, sections were then incubated with 4’, 6’-diamino-2-phenylindole (DAPI) for 2 min to stain nuclei. The labeled sections were then examined by a confocal microscope (Model LSM 510; Carl Zeiss Canada Ltd., Toronto, Ontario), equipped with appropriate lasers. In negative controls, the two primary antibodies were replaced with non-immune rabbit serum and mouse IgG. The primary antibodies have been used previously for GH and GHR localization in human tissues [3,13,14].

To confirm the identity of cells in the GCL, we used C-20 (Santa Cruz Biotechnology Inc., Santa Cruz, CA), a polyclonal antibody, against γ-synuclein [15,16]. This antibody was used at a dilution of 1:50 for 2 h at room temperature, and its specificity was confirmed by pre-absorption with the blocking peptide (C-20P) supplied by the company. Double labeling for GH and synuclein was performed by initially incubating sections overnight in GH antiserum followed by incubation with anti-synuclein.

For the TUNEL technique, sections were dewaxed and washed in PBS before they were treated with 2.5 μg/ml proteinase K for 10 min at room temperature. Sections were then treated for 5 min with a TdT buffer that consisted of 30 mM Trizma base, 140 mM sodium cacodylate, and 1 mM cobalt chloride (pH 7.2). Tissue was incubated with the reaction mixture for 1.5 h at 37 °C in a humid chamber, using the kit from Roche Molecular Biochemicals (Laval, Quebec) at a volume of 20 μl per sample. A volume of 100 μl contained: double distilled water, 81 μl; TdT buffer, 6.5 μl; cobalt chloride, 3.26 μl; biotin-16-dUTP stock, at a concentration of 1 nmol/µl, 1.86 μl; dUTP, 5.5 μl; and TdT, at a concentration of 10 units/μl, 2 μl. Samples were then washed in PBS three times for 5 min each, and blocked with 3% skimmed milk in PBS containing 0.5% Tween-20 for 30 min. Samples were washed in PBS three times for 5 min each, and the fluorochrome, Streptavidin-FITC, was added at a concentration of 1:200, for 1 h at room temperature. Following 3 washes in PBS for 10 min each, the samples were taken through the immunocytchemistry procedure described above, using Alexa-Fluor 594 as the secondary antibody, and mounted on slides with Vectashield (Vector Laboratories Canada Inc., Burlington, Ontario). All cell counting was performed by manually counting each cell seen in the confocal images. All images of the retina were obtained from within approximately 5 mm of the optic nerve head. Sample sizes (n) are the combined numbers of cells from all three retina samples.

## Results

**Figure 1:**
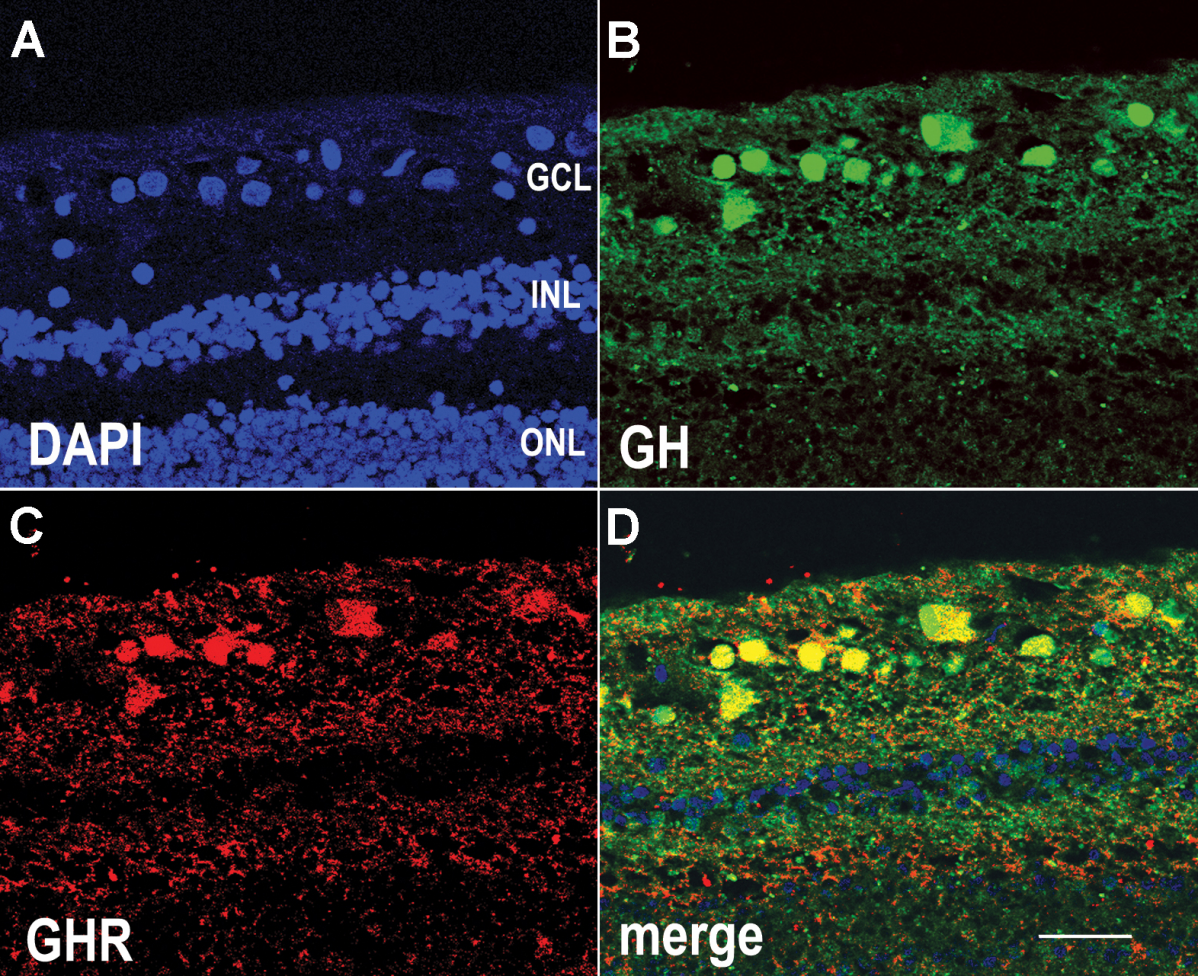
A human retina section immunocytochemically labeled for GH and GHR. **A:** DAPI labeling (blue) shows the position of all nuclei in the section. **B:** Growth hormone (GH) antiserum (green) labels cells in the ganglion cell layer (GCL). **C:** GH receptor (GHR) antibodies (red) label cells in the GCL. **D:** When panels **B** and **C** are merged, it is clear that GH and GHR are co-localized in the same cells (yellow). Abbreviations: inner nuclear layer (INL); outer nuclear layer (ONL). Scale bar equals 40 μm.

**Figure 2:**
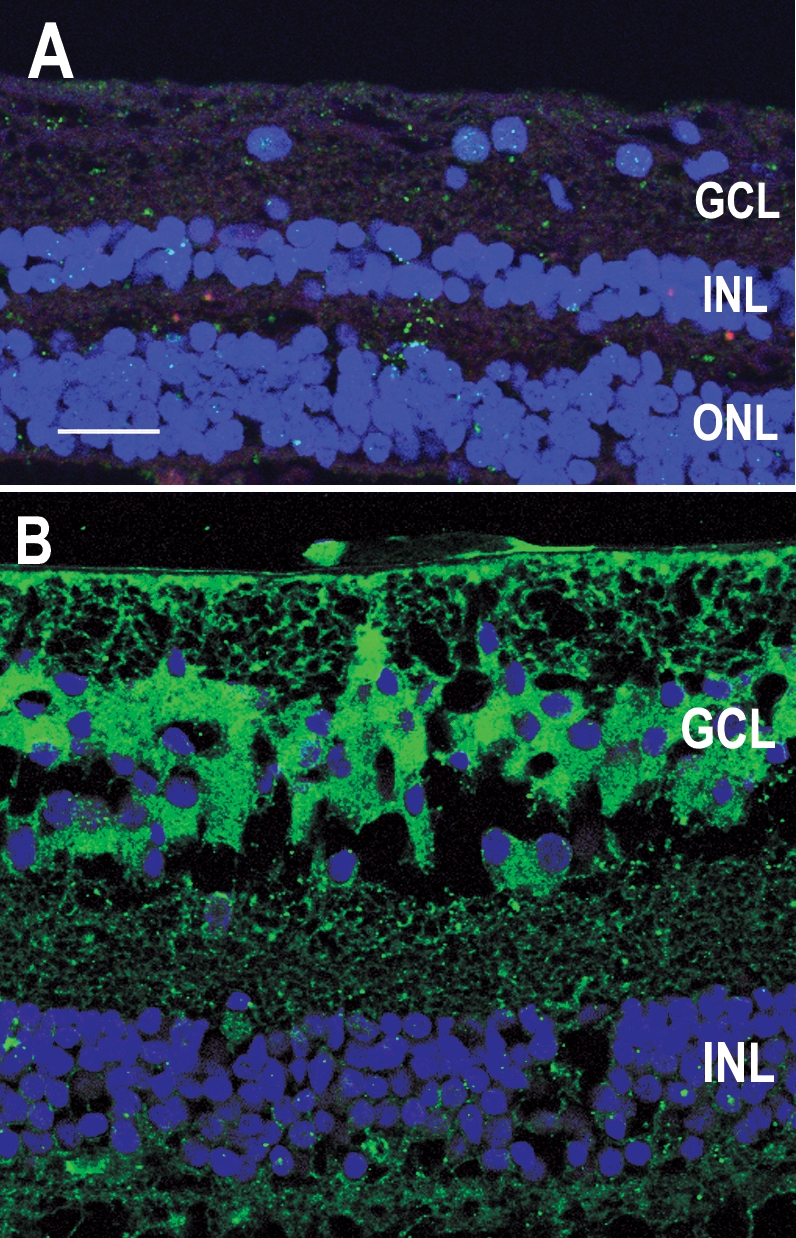
Human retina sections showing an immunocytochemical control and γ-synuclein labeling. **A:** This image shows immunocytochemical labeling of a section in which growth hormone (GH) antiserum was replaced by rabbit serum and GH receptor (GHR) antibodies were replaced by mouse IgG. DAPI labeling (blue) shows the position of all nuclei in the section. **B:** This is an oblique section through the retina, labeled with an antibody against γ-synuclein (green) to identify retinal ganglion cells (RGCs) in the ganglion cell layer (GCL). The majority of cells in the GCL show cytoplasmic labeling for synuclein. DAPI labeling (blue) shows the position of all nuclei in the section. Abbreviations: inner nuclear layer (INL); outer nuclear layer (ONL). Scale bar equals 40 μm.

Immunocytochemical localization of GH and GHR in human retina sections (Figure 1) indicates that both are preferentially located in cells of the GCL, where they colocalized (yellow, in the merged image) in the nucleus as well as cytoplasm of many cells. Little or no GH was expressed in other cell layers. Analysis of the numbers of GH-positive or GHR-positive cells in the GCL from three different retina samples indicated that, overall, approximately 35% of cells in the GCL were GH-positive (n=56 cells), while a somewhat smaller number were GHR-positive. Because the GHR signal was weaker than the GH signal, the apparently lower number of GHR-positive cells may be due to the lower sensitivity of the technique. Cells of the inner and outer nuclear layers (Figure 1) were not immunoreactive for GH or GHR. When the primary antibodies were replaced with rabbit serum (for GH antiserum) or mouse IgG (for GHR antiserum), the immunoreactivity was abolished (Figure 2A).

**Figure 3:**
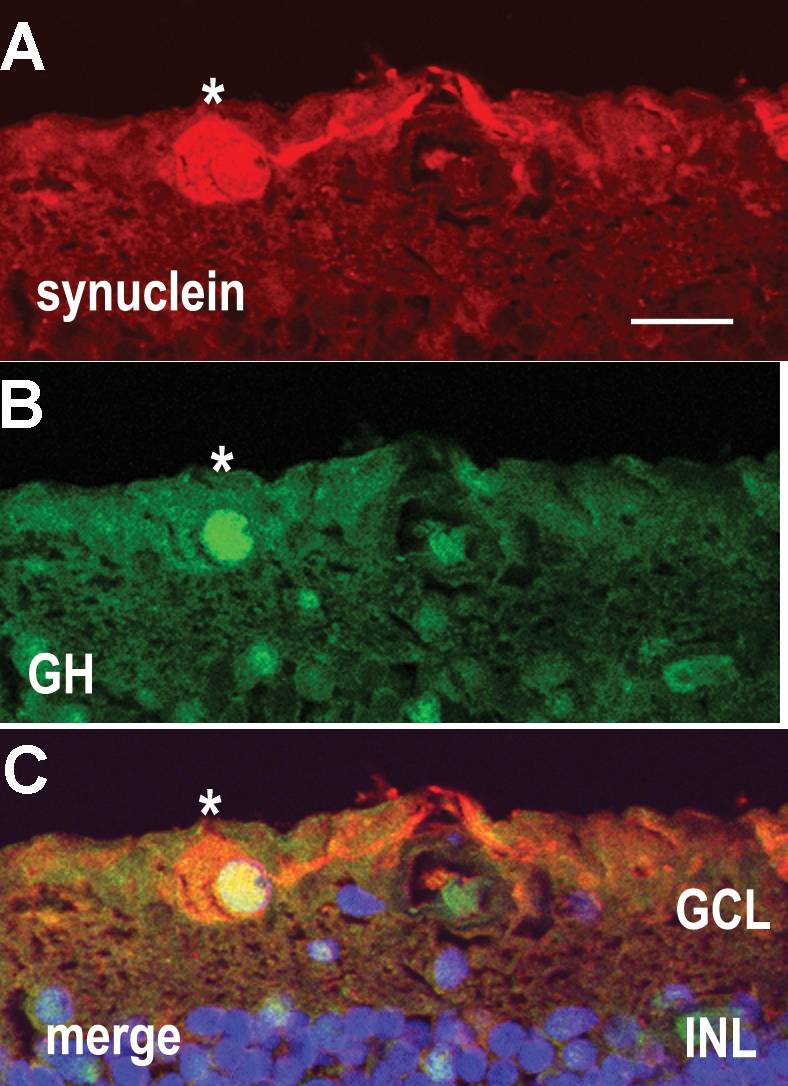
A human retina section double-labeled for γ-synuclein and GH. **A:** γ-synuclein antibodies (red) label a retinal ganglion cell (RGC) in the ganglion cell layer (GCL). **B:** growth hormone (GH) antiserum (green) labels a nucleus in the GCL. **C:** The merged image of panels **A** and **B** shows that GH localizes to the same cell as γ-synuclein, an RGC marker, (asterisks) indicating that this GH-containing cell is an RGC. DAPI labeling (blue) shows the position of all nuclei in the section. Abbreviations: ganglion cell layer (GCL); inner nuclear layer (INL). Scale bar equals 20 μm.

To confirm the identity of cells in the GCL, we labeled sections with an antibody against γ-synuclein, a marker for RGCs [15,16]. The majority of cells in the GCL were preferentially labeled by this antibody, largely in the cytoplasm (Figure 2B). DAPI labeling was used as a cell marker of all cells. At least 95% (n=200 cells) of the cells in the GCL were labeled by this antibody. When the blocking peptide supplied by the manufacturer was added to the antibody before its use, immunoreactivity was abolished. Double labeling sections for γ-synuclein (red) and GH (green) showed that GH was localized to RGCs (Figure 3; asterisks).

**Figure 4:**
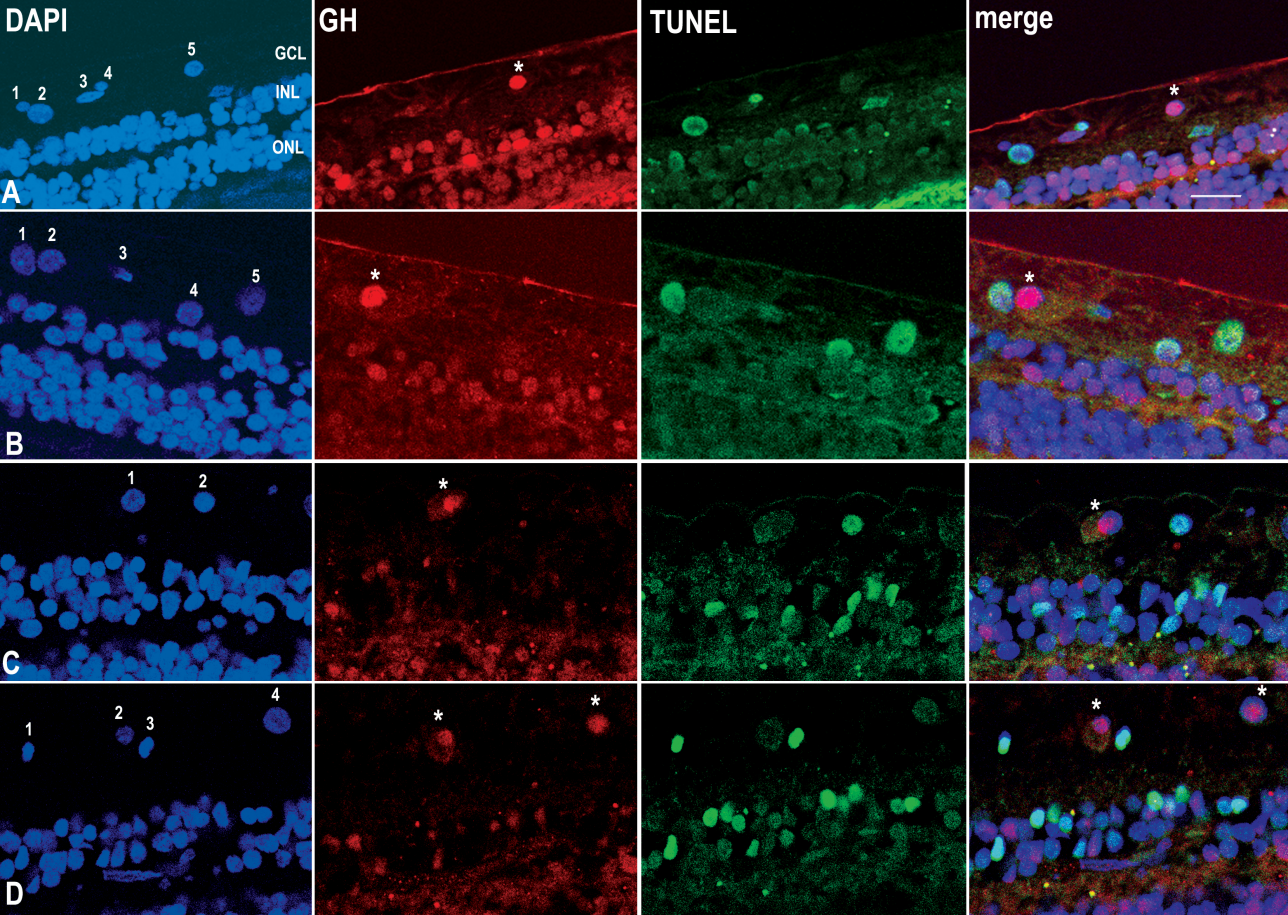
Sections of human retina immunocytochemically labeled for GH and dying cells. **A:** A section from a 74 year-old male labeled with DAPI (blue) for nuclei, growth hormone (GH) antiserum (red; asterisk); and TUNEL (green) for apoptotic nuclei. **B:** Another section from the same individual, labeled in the same way as **A**. **C:** A section from an 81 year-old male, labeled in the same way as **A**. **D:** A section from a 69 year-old male, labeled in the same way as **A**. With respect to cells in the ganglion cell layer (GCL), in the merged images, non-apoptotic and GH-positive nuclei appear red (asterisks) and correspond to nuclei numbered: **A** 5, **B** 2, **C** 1, **D** 2, and **D** 4. Apoptotic and GH-negative nuclei **A** 2, **A** 4, **B** 1, **B** 4, **B** 5, **C** 2, **D** 1, and **D** 3, appear blue-green. Non-apoptotic and GH-negative nuclei **A** 1, **A** 3, and **B** 3 appear blue. Abbreviations: inner nuclear layer (INL); outer nuclear layer (ONL). Scale bar equals 20 μm.

Double labeling cells with GH and TUNEL for apoptotic DNA fragmentation (Figure 4) showed that positive immunoreactivity for GH always correlated with the absence of TUNEL labeling. Overall, based on three different retina samples, 46.4% of cells in the GCL were TUNEL-positive (n=56 cells). Of these, 100% were GH-negative (n=35 cells). Of the remaining TUNEL-negative cells, 32% were GH-negative (n=48 cells).

In Figure 4, it can be seen that cells labeled A5, B2, C1, D2, and D4 (asterisks) are all GH-positive (red) and TUNEL-negative (red nuclei in the merged images). By contrast, cells A2, A4, B1, B4, B5, C2, D1, and D3 are TUNEL-positive (green) but GH-negative (blue-green nuclei in the merged images). Approximately 18% of cells are both TUNEL-negative and GH-negative (A1, A3, and B3; blue in the merged images).

## Discussion

There are limited experimental options available for use in attempts to establish a functional role for endogenous GH in adult human RGCs. In this study, we chose to correlate the presence of immunoreactive GH with a marker of cell death in the GCL from sectioned human retinas. We found that cells of the GCL that contained TUNEL-positive nuclei never expressed GH immunoreactivity in their nucleus or cytoplasm. Of the remaining cells that contained TUNEL-negative nuclei, two-thirds had GH in the cytoplasm as well as nuclei. Labeling of sections with the RGC marker synuclein [15,16], indicated that at least 95% of the cells in the GCL were RGCs, leading us to conclude that the majority of the cells that we have examined in the GCL are RGCs and not cells displaced from other layers of the retina [17]. This result therefore provides us with circumstantial evidence that endogenous GH in human RGCs may have a role in cell survival, and is consistent with our previous experimental studies on chick RGCs showing that GH has anti-apoptotic properties both in vivo and in vitro in these cells during development [6,8,9].

One third of the TUNEL-negative RGCs apparently did not express GH. We conclude that GH is sufficient, but not necessary, for RGC survival. Presumably, such cells may receive survival signals from other molecules, since GH is by no means the only neuroprotective molecule present in the retina [18].

We found that 46% of the cells in the GCL of our samples were TUNEL-positive. This number of apoptotic cells may be attributed not only to natural cell death in this layer, but also possibly to postmortem cell death, since fixation of the specimens was performed between 2 and 13 h postmortem. However, evidence from examination of retinas after optic nerve transection [19] indicates that RGCs survive for several days after this insult. It is likely that GH is able to rescue RGCs irrespective of the cause of cell death in the GCL, whether owing to disease or whether it is post-mortem, since our in vitro results with chick RGCs show that GH is also able to protect these cells from apoptosis after axotomy [19] that is performed to culture the cells [6,8,9].

That GH and GHR are mostly colocalized to the nuclei is not unexpected; it has long been known that both GHR and GH are associated with the nucleus [20]. Technically, we performed the more complex TUNEL procedure before the immunocytochemistry. In contrast to GH, immunoreactivity for GHR did not survive the TUNEL procedure, presumably because of the weaker immunoreactivity of GHR in comparison with GH. It is unlikely that the TUNEL procedure inhibits subsequent GH immunoreactivity, because the GH immunoreactivity clearly survives the proteinase-K treatment (compare cell A4 with A5; and B1 with B2), which is the only step in the TUNEL procedure likely to interfere with GH immunoreactivity.

On the basis of these results, the question arises as to whether or not patients with low systemic GH levels experience retinal cell death or other retinopathies. Bearing in mind that systemic levels of pituitary GH may not be relevant to the ocular levels of locally produced GH [7], the literature suggests that such patients may indeed exhibit ocular dysfunction [21]. Dysfunctions related to GH deficiency include: reduced retinal vascularization, optic disc and optic nerve hypoplasia, Jacobsen syndrome, Cockayne syndrome, and a rare form of retinitis pigmentosa (RHYNS syndrome) characterized by hypopituitarism, GH deficiency, and skeletal dysplasia [21].

### Conclusion

We conclude that in the human retina, the presence of GH in the RGCs correlates with the survival of these cells. This conclusion is consistent not only with our previous experimental results [6,8,9], but also with a growing literature indicating the general anti-apoptotic and neuroprotective effects of GH [22] in diverse cell types including: myoblasts [23], myocytes [24], neonatal brain neurons [25], and cells of the immune system [26].
